# Ratphones: An Affordable Tool for Highly Controlled Sound Presentation in Freely Moving Rats

**DOI:** 10.1523/ENEURO.0028-23.2023

**Published:** 2023-05-24

**Authors:** Mafalda Valente, Juan R. Castiñeiras-de Saa, Alfonso Renart, Jose L. Pardo-Vazquez

**Affiliations:** 1 Champalimaud Research, Champalimaud Centre for the Unknown, 1400-038 Lisbon, Portugal; 2 Neuroscience and Motor Control Group, Centro Interdisciplinar de Química e Bioloxía (CICA), Universidade da Coruña, 15071 A Coruña, Spain

**Keywords:** behaving animals, headphones, interaural level differences, rodents, sound presentation

## Abstract

Encoding and processing sensory information is key to understanding the environment and to guiding behavior accordingly. Characterizing the behavioral and neural correlates of these processes requires the experimenter to have a high degree of control over stimuli presentation. For auditory stimulation in animals with relatively large heads, this can be accomplished by using headphones. However, it has proven more challenging in smaller species, such as rats and mice, and has been only partially solved using closed-field speakers in anesthetized or head-restrained preparations. To overcome the limitations of such preparations and to deliver sound with high precision to freely moving animals, we have developed a set of miniature headphones for rats. The headphones consist of a small, skull-implantable base attached with magnets to a fully adjustable structure that holds the speakers and keeps them in the same position with respect to the ears.

## Significance Statement

Presenting sensory stimulation reliably is critical in many experimental paradigms. Different methods have been used to accomplish this goal in different sensory modalities, but it has proven difficult to control sound presentation in small species, such as rats and mice. In this work, we present the Ratphones, a set of miniature headphones, and provide all necessary information for building, adjusting, and using the Ratphones to reliably present auditory stimulation to freely moving rats.

## Introduction

Processing sensory inputs is crucial for understanding the environment and adjusting behavior accordingly. In addition to psychophysics, in which sensory evidence is critical, sensory stimuli are used in many paradigms within behavioral neuroscience. For these experiments to be valid and reliable, it is key to ensure a high degree of control over the physical properties of the stimulation that reaches the sensory organs, so that that they can be accurately replicated. Moreover, precise stimulus control is critical for the experimenter to be able to interpret neural variability and its relationship with behavior; only if one can accurately repeat the same stimulus, can a distinction between internal and external neural variability be made.

In visual experiments, this requirement has been fulfilled mostly by using eye-tracking systems in head-fixed (or head-restrained) subjects (Britten et al., 1993), but also by using head-mounted eye-trackers (Cognolato et al., 2018). Recently, a magnetic eye-tracking system that can be used in both head-fixed and freely moving mice has been developed (Payne and Raymond, 2017). For olfactory and tactile stimulation, researchers have developed high-precision devices for this purpose (Romo et al., 2002; Kepecs et al., 2006). In the auditory modality, there have been two main strategies. On the one hand, in species with relatively large heads, such as monkeys (Schroeder et al., 2001; Fishman and Steinschneider, 2009) or ferrets (Nodal et al., 2010; Keating et al., 2013), sound presentation can be controlled by using headphones. On the other hand, for smaller animals, such as rats and mice, the sound can be reliably delivered by using head-fixed (Joachimsthaler et al., 2014) or anesthetized (Yao et al., 2013) preparations. However, to our knowledge, delivering sound under strictly controlled conditions to freely moving rodents is a challenge that has been only partially solved so far in rats by chronically implanting a plastic structure into which the speakers were screwed before each behavioral session (Otazu et al., 2009).

We have designed the Ratphones, an affordable set of miniature headphones that consists of a small, skull-implantable base attached with magnets to a fully adjustable structure that holds the speakers and keeps them in the same position with respect to the ears. With the Ratphones, the experimenter only needs to implant a small base, which is less disruptive for the animals than implanting the whole structure except for the speakers, and the headphones are attached to this base with magnets, thus avoiding the need to screw (and unscrew) the speakers before (and after) every behavioral session.

These headphones allow the experimenter to control independently the sound delivered to the two ears, which is especially important for studying sound localization, where the interaural level difference (ILD) of the sound is the main cue used by the auditory system to extract azimuth in the horizontal plane (Wesolek et al., 2010). The speakers we chose are good for high frequencies (up to 40 kHz) and can deliver pure tones and narrowband noise. Thus, the Ratphones can be used in most experiments requiring highly controlled sound presentation. However, the speakers are limited in terms of sound intensity [maximum, 79 dB SPL (measured in 0.1 m distance)] and may not be the best option for experiments demanding very high sound intensities.

## Design Requirements

The main functional requirements behind this design were to have a precise, reliable, and robust relative positioning between the speaker and the pinna, while at the same time allowing flexibility to adjust this positioning to the variations between base positioning and pinna location on each individual animal. To achieve this, (1) the design contains movable pieces that can be adjusted on the anteroposterior, mediolateral, and dorsoventral axes; and (2) the procedure to configure the Ratphones consists of a first step in which the base is implanted, and a second step in which the pieces are adjusted for each individual animal under anesthesia and glued in their final configuration for each rat.

Another critical design requirement for the Ratphones was to use them in behaving animals, as opposed to anesthetized or head-fixed preparations. Mostly because of this requirement, we decided to use external headphones instead of placing them in the ear. In-ear headphones in principle afford a higher degree of control, as sounds not coming from the speakers are blocked. They also allow pure monaural stimulation (i.e., one is sure that each ear only hears the sound from its corresponding speaker), which, as previously mentioned, is important for controlling ILD. They have, however, the important drawback that they are much more invasive and uncomfortable for the subject, which, especially in a behavioral context where one has to fit them on every behavioral session, is critical. If the animal starts every session stressed and uncomfortable, it will interfere with the behavioral readouts of the sensory measurements the experimenter is trying to perform. A second important drawback for internal headphones, based on human subjective experience, is the sensitivity of this configuration to slight adjustments in the positioning of the earphone (and supporting sound-isolating material) relative to the inner pinna. A bad seal can completely compromise accurate sound delivery, and the rats cannot report on a bad seal. For all these reasons, we decided to use an external design with close placement. Regarding the downsides, we tested explicitly that monaural contamination is small compared with behavioral ILD sensitivity, but we would in general recommend performing experiments in a sound-isolation box, where the possibility of interference because of a lack of the seal provided by an internal design is minimized.

In this work, we provide all necessary information for building, adjusting, and using the Ratphones to reliably present auditory stimulation to freely moving rats. Empirical data obtained with the Ratphones can be found in the study by Pardo-Vazquez et al. (2019).

## Materials and Methods

**Figure 1.**
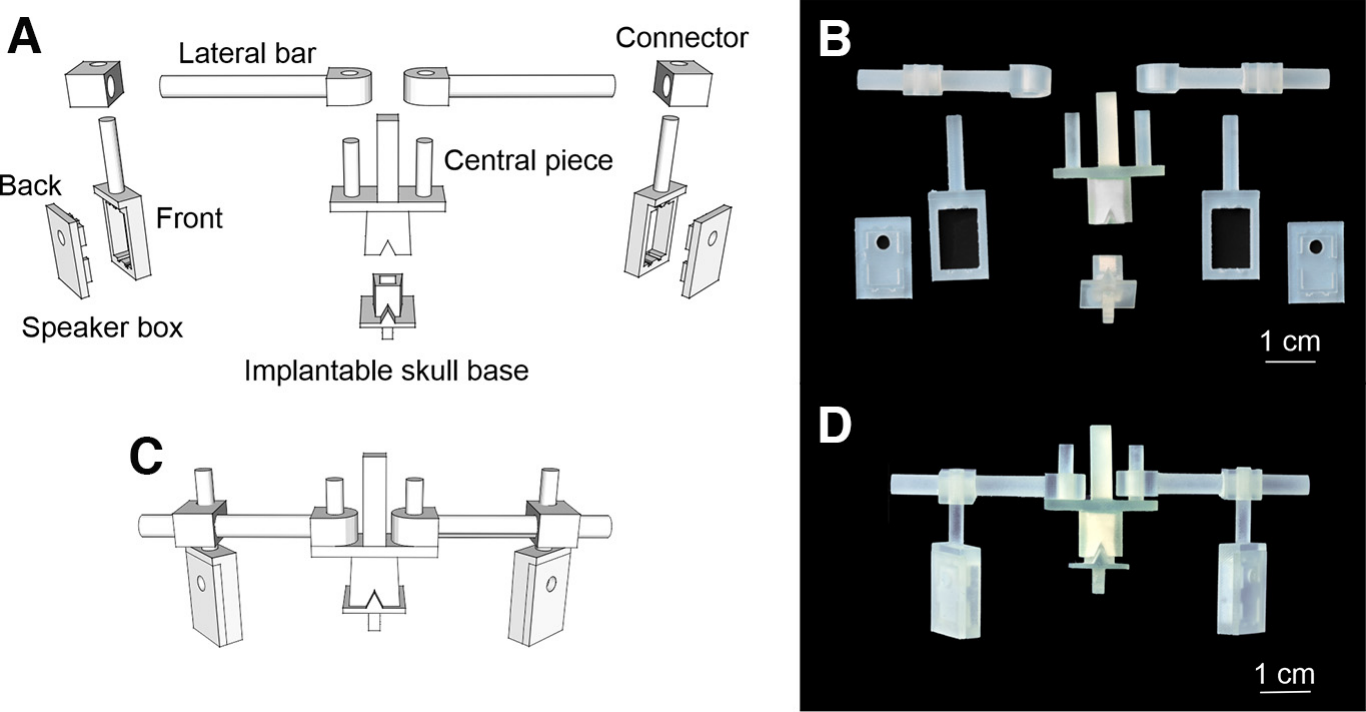
Ratphones 3D design and 3D-printed resin pieces. ***A***, Set of 3D-printable parts. ***B***, Set of resin-printed parts. ***C***, ***D***, Front view of all the parts assembled to form the final structure that holds the speakers, in the 3D model and printed, respectively.

The Ratphones consist of a small, chronically implantable base and a set of movable parts (Fig. 1*A*,*B*) that can be put together to form a structure that holds the headphones in the desired position with respect to the ears (Fig. 1*C*,*D*). The structure is attached to the base using magnets. All parts, except for the magnets and speakers (Table 1), can be 3D printed using the stereolithography files (stls) we provide in https://github.com/JosePardoVazquez/RatHeadphones. These parts have been designed to be printed using stereolithography (Fig. 1*B*,*D*), but can be easily adapted to other 3D-printing methods. In its current form, the speaker box is designed to be used with a specific receiver model (Table 1), but it can be easily redesigned to fit other models without modifying the other parts. Below, we describe the procedure we used for implanting the base and adjusting the headphones. All procedures were reviewed and approved by the animal welfare committee of the Champalimaud Centre for the Unknown and approved by the Portuguese Direcção Geral de Veterinária (reference #0421/000/000/2019).

**Table 1:** List of parts needed to build a set of Ratphones

Parts	Description
3D-printed pieces (stls files provided)	Implantable skull base
	Central piece
	Lateral bar (×2)
	Speaker box, front (×2)
	Speaker box, back (×2)
	Lateral bar to speaker box connector (×2)
Magnets	B222 (K&J Magnets, Inc.) one-eighth × one-eighth × one-eighth inch thickness; grade N42, nickel-plated magnetized through thickness
	B3301 (K&J Magnets, Inc.) three-sixteenth × three-sixteenth × one-thirty-second inch thickness; grade N42, nickel-plated magnetized thru thickness
Speakers	Knowles active receiver (MOD 2403260 00031; “Petra”)
Connector	Connector header through hole 3 position 0.100 inch (2.54 mm)

### Base implant surgery

**Figure 2.**
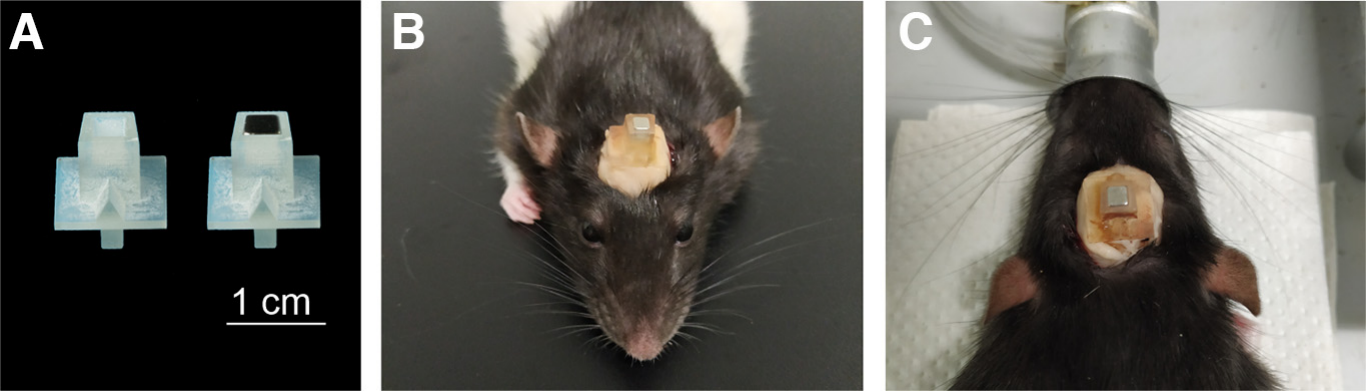
***A***, Skull base 3D printed in resin, without and with the magnet in place. ***B***, ***C***, Front and rear view of a skull base chronically implanted.

Anesthesia was induced by inhalation of isoflurane at a concentration of 5% (oxygen at 2 L per min) and maintained by an injection of ketamine/xylazine (0.1 ml/100 g, i.p.). More isoflurane was occasionally administered for longer surgeries if the animal exhibited signals of pain or discomfort. The animal was shaved and fixed to the stereotaxic frame, and eye ointment was applied to the eyes. Lidocaine (0.2 ml) was injected subcutaneously at the incision site before the incision was made, for local anesthesia. The skin was cleaned using iodine, an incision (∼2 cm in length) was practiced along the midline, and the skin was displaced laterally, exposing the surface of the skull. After cleaning the top region of the skull by blunt dissection, four drilling holes were made and titanium screws (length, 3 mm; thread diameter, 1 mm) were attached to the skull, allowing for most of their length to remain outside. Cement was poured on top of these screws, ensuring it reached the space between the screws and the skull for a secure attachment [using a strong dental adhesive, such as Super-Bond (Sun Medical), it might be possible to firmly implant the base without screws; this cement has shown high tolerance, resistance, and durability for chronic implants in different species, including mice (Lohse et al., 2021) and ferrets (Nodal et al., 2010)]. A small cube-shaped magnet (Table 1) was placed inside the resin base (Fig. 2*A*), which was then placed on top of the cement layer, and more cement was added around the lower part of the base until it was covered. The displaced skin was then stitched around the base, only allowing the necessary structure for the attachment of the headphones to remain visible (Fig. 2*B*,*C*). Antibiotics (8 mg/kg, s.c.; cefovecin, Convenia) and analgesics (5 mg/kg, s.c.; carprofen, Rimadyl) were administered after the surgery. The base (with the magnet) weighs ∼0.9 g, but together with the cement it weighs ∼2.4 g.

### Individual adjustment of the Ratphones

**Figure 3.**
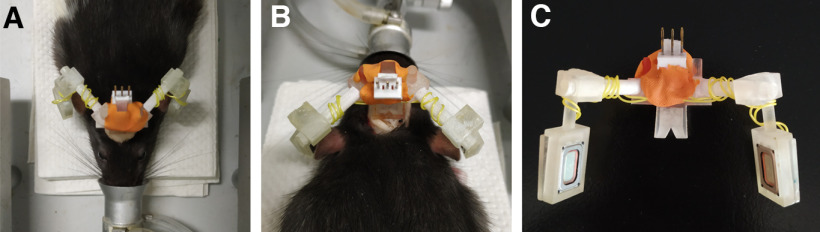
***A***, ***B***, Front and rear view of a set of Ratphones adjusted under anesthesia. ***C***, Set of Ratphones assembled and glued together, including the speakers, cables, and connector.

This procedure was performed 1 week after the base implant surgery, during which the animal was allowed to recover with free access to water and food. Under anesthesia (induced and maintained with isoflurane at 4% and 2.5%, respectively), the structure with all pieces temporarily assembled, including the speakers and their connections, was placed on the implanted base and the different angles between the pieces were adjusted so that the speakers were placed at ∼5 mm from the opening of the ear of the animal. The pieces were then fixed with cyanoacrylate, removed from the animal, and covered with flexible silicone rubber (Sugru), providing extra fixation to the resin pieces and protection to the electrical cables that connect the speakers. Initially, we covered most of the structure of the headphones with flexible silicon rubber (Pardo-Vazquez et al., 2019, their Supplementary Fig. 1), but lately we have been covering only the central piece (Fig. 3), reducing the weight of the Ratphones while keeping a strong attachment in the part that supports more tension when attaching/detaching them. A magnet (Table 1) was glued in the bottom of the structure to attach it to the implanted base during the behavioral sessions. A three-pin male connector (Table 1) was affixed to the part of the 3D-printed structure. Before each behavioral session, a standard three-wire sound cable—soldered to a matching three-pin female connector—was plugged to the male connector. A passive commutator, attached to the ceiling of the behavioral box, was used to avoid tangling. The headphones weigh 7.22 g without flexible silicone rubber and 12.8 g with a full coverage of this material.

We think that it should be possible to scale the Ratphones down to be used in freely moving mice. The 3D parts can be easily scaled; there are miniature neodymium magnets that are strong enough to keep the headphones attached to the base (e.g., a 1.6 × 1.6 × 1.6 mm neodymium cube weighs 0.06 g and can hold 90 g); and there are speakers that are light (0.6 g) and small (diameter, 10 mm) enough to be used in mice.

### Behavioral and sound attenuation tests

The Ratphones were used in freely moving rats performing a sound lateralization task (Pardo-Vazquez et al., 2019). The arena consisted in a standard Coulbourn Instruments modular box (30 × 25 × 30 cm) equipped with three nose-pokes, one of them with a water delivery system. Before each behavioral session, the rat was placed in the box and a set of individualized Ratphones was brought near to the implanted base, until the magnets were attached, without restraining the movement of the animal. The Ratphones were plugged, through a standard sound cable, to a real time processor (RP2 by Tucker-Davis Technologies) that controlled the behavioral task, including presenting the sound and recording the responses of the animal. To avoid tangling, the cable was attached to a passive commutator.

Since we decided to minimize any physical contact between the speakers and the pinnae, it is expected that some residual sound from one speaker will reach the contralateral ear. We addressed this empirically, by playing cosine-ramped (10 ms) broadband noise (5–20 kHz) at 65, 70, and 75 dB SPL from one speaker and recording the sound with the microphone placed by the contralateral ear canal. The noise was independently generated for each presentation using a RP2 module at a sample rate of 50 kHz. The speakers were calibrated using a Brüel & Kjær Free-field one-quarter inch microphone placed in front of the speaker, 5 mm apart. We found that the head plus near-field positioning of the speaker attenuates the sound by ∼22 dB (Pardo-Vazquez et al., 2019, data published in their supplementary information). Since the just noticeable difference for lateralization of sound in this task is 2.2 dB (Pardo-Vazquez et al., 2019), this suggests that level differences played through the Ratphones are an accurate approximation of actually experienced level differences (relative to the behavioral accuracy for sound lateralization in rats), which validates the use of the Ratphones for psychophysical testing of sound lateralization.

## Synthesis

Reviewing Editor: Matthew Grubb, King’s College London Decisions are customarily a result of the Reviewing Editor and the peer reviewers coming together and discussing their recommendations until a consensus is reached. When revisions are invited, a fact-based synthesis statement explaining their decision and outlining what is needed to prepare a revision will be listed below. The following reviewer(s) agreed to reveal their identity: Katarina Leao.

Reviewer 1:

This manuscript describes ratphones, a tool for binaural sound stimulation in freely moving rats and provides description of 3D printed parts.

Some minor concerns raised are the following:

1) How much does the complete implant and attachment weigh?

2) Can the rat scratch it off (etc., how strong is the magnet)? Could the rat get stuck with the claws in the yellow cables?

At line 128 the authors write “The pieces were then fixed with cyanoacrylate, removed from the animal and covered with Sugru®, providing extra fixation to the resin pieces and protection to the electrical cables that connect the speakers (Figure 3).”

However, the cables are not covered in figure 3, but are so in their previous publication Supplementary figure 1A (Pardo-Vazquez et al., 2019). I would suggest to use a photo similar to the one already published.

Also, could the cables be covered with standard heat-shrink tubes? This information should be provided in the current manuscript.

3) At line 127 the authors write “the speakers were placed at about 5 mm from the opening of the animal’s ear” and then at line 134 “ we decided to avoid any physical contact between the speakers and the pinnae”. This seems not possible if the speaker is 5mm from the opening of the ear.

In figure 3B it looks reasonable that the pinna will touch the speakers if the rat moves the ears sideways or up. What is the drawback from possible sensory stimulation of the ears? This could be discussed more in the current manuscript. For example, the dorsal cochlear nucleus integrates auditory and sensory input, and its known that sensory perception from the pinnae (shown in cats) can contribute to sensory integration in the dorsal cochlear nucleus.

4) The authors suggest that the ratphones are useful for studying processing of interaural level differences (ILD) for sound localization. Could the authors comment on the usefulness for other experiments, such a loud noise exposure or fear behavior studies?

What are the limitations of the speakers in respect to loudness and frequency range? The manuscript states in line 136 that “broadband noise (5-20 kHz) at 65, 70 and 75 dB SPL” was tested. No calibration of speakers was described in this manuscript, so how can the authors confirm that indeed 5-20kHz was delivered? Many speakers with the stated range up to 20kHz actually only reach max 18kHz, in my experience. Also, can these speakers deliver narrow band noise? Pure tones? What is the sound pressure level min - max range? Providing this information would be useful for attracting the broader auditory community to using the ratphones as described here.

5) It would be nice if the authors could comment on the scalability for the ratphones for use in freely moving mice, in respect to size of speakers and weight for example.

Reviewer 2:

This manuscript reports the technical details necessary to produce a set of detachable headphones for rats. The novelty of it is in jeopardy because of the previous publication by the authors on which such device was used and described (Pardo-Vazquez et al. 2019). Also, similar headphone setups have been published for different species (rats: Otazu et al., 2009; ferrets: Nodal et al. 2010). Therefore, the advance in the field provided by this manuscript is just slightly incremental.

Setting aside the novelty of the manuscript, I have some comments to improve it.

Main comments

The introduction is biased on the utility of the present setup for ILD discrimination. I think the main advantage is that it allows the presentation of the stimulus without the need of previous conditioning to ensure the correct positioning regarding an external speaker for repeatability of the stimulus presentation. This point is made by Otazu et al 2009, a reference should be included as it describes also a different headphone system for rats in which only the actual speakers were detachable.

The use of bone screws to fix the implant maybe is not the most refined practice as new products used in dental practice (e.g. Super-bond) that exhibit very good adhesion to bone and plastic can be used. The tolerance and resistance and durability of such product has already been reported in several species (e.g. Nodal et al 2010, Lohse et al 2021)

Minor

The reference by Otazu et al 2009 contradicts the statement in line 44.

I would replace internal headphones by in-ear headphones.

Line 96 the actual committees approving the work should be listed

The drugs used should be listed and doses stated according to the active substance not by their commercial names, as those can vary from country to country (line 117).

Same for Sugru line 129

In line 30 “noise” maybe is not the best choice of words when describing a headphone system as it is unclear if when referring to internal and external noise they are referring to an actual noise produced by the animals vs the noise produced by the headphones or they are referring to neural noise not related to the actual stimulus as neural variability is used.

Line 37 typo an “e” is missing

Figure 1 legend instead of attached used assembled.

Table 1 include the electrical connector used for the headphones

Also details of the cable used to tether the animals and drive the speakers and of the arena in which those headphones have been tested should be included to have an idea of tethering issues.

Nodal FR, Keating P, King AJ. Chronic detachable headphones for acoustic stimulation in freely moving animals. J Neurosci Methods. 2010 May 30;189(1):44-50. doi: 10.1016/j.jneumeth.2010.03.017. Epub 2010 Mar 25. PMID: 20346981; PMCID: PMC2877876.

Lohse M, Dahmen JC, Bajo VM, King AJ. Subcortical circuits mediate communication between primary sensory cortical areas in mice. Nat Commun. 2021 Jun 24;12(1):3916. doi: 10.1038/s41467-021-24200-x. PMID: 34168153; PMCID: PMC8225818.

Otazu GH, Tai LH, Yang Y, Zador AM. Engaging in an auditory task suppresses responses in auditory cortex. Nat Neurosci. 2009 May;12(5):646-54. doi: 10.1038/nn.2306. Epub 2009 Apr 12. PMID: 19363491; PMCID: PMC4084972.

Pardo-Vazquez, J.L., Castiñeiras-de Saa, J.R., Valente, M. et al. The mechanistic foundation of Weber’s law. Nat Neurosci 22, 1493-1502 (2019). https://doi.org/10.1038/s41593-019-0439-7
